# Validity and Cultural Generalisability of a 5-Minute AI-Based, Computerised Cognitive Assessment in Mild Cognitive Impairment and Alzheimer's Dementia

**DOI:** 10.3389/fpsyt.2021.706695

**Published:** 2021-07-22

**Authors:** Chris Kalafatis, Mohammad Hadi Modarres, Panos Apostolou, Haniye Marefat, Mahdiyeh Khanbagi, Hamed Karimi, Zahra Vahabi, Dag Aarsland, Seyed-Mahdi Khaligh-Razavi

**Affiliations:** ^1^Cognetivity Ltd, London, United Kingdom; ^2^South London & Maudsley NHS Foundation Trust, London, United Kingdom; ^3^Department of Old Age Psychiatry, King's College London, London, United Kingdom; ^4^School of Cognitive Sciences, Institute for Research in Fundamental Sciences (IPM), Tehran, Iran; ^5^Department of Stem Cells and Developmental Biology, Cell Science Research Centre, Royan Institute for Stem Cell Biology and Technology, ACECR, Tehran, Iran; ^6^Tehran University of Medical Sciences, Tehran, Iran

**Keywords:** computerised cognitive assessment, integrated cognitive assessment, machine learning, artificial intelligence, mild cognitive impairment, mild Alzheimer disease

## Abstract

**Introduction:** Early detection and monitoring of mild cognitive impairment (MCI) and Alzheimer's Disease (AD) patients are key to tackling dementia and providing benefits to patients, caregivers, healthcare providers and society. We developed the Integrated Cognitive Assessment (ICA); a 5-min, language independent computerised cognitive test that employs an Artificial Intelligence (AI) model to improve its accuracy in detecting cognitive impairment. In this study, we aimed to evaluate the generalisability of the ICA in detecting cognitive impairment in MCI and mild AD patients.

**Methods:** We studied the ICA in 230 participants. 95 healthy volunteers, 80 MCI, and 55 mild AD participants completed the ICA, Montreal Cognitive Assessment (MoCA) and Addenbrooke's Cognitive Examination (ACE) cognitive tests.

**Results:** The ICA demonstrated convergent validity with MoCA (Pearson r=0.58, p<0.0001) and ACE (r=0.62, p<0.0001). The ICA AI model was able to detect cognitive impairment with an AUC of 81% for MCI patients, and 88% for mild AD patients. The AI model demonstrated improved performance with increased training data and showed generalisability in performance from one population to another. The ICA correlation of 0.17 (*p* = 0.01) with education years is considerably smaller than that of MoCA (*r* = 0.34, *p* < 0.0001) and ACE (*r* = 0.41, *p* < 0.0001) which displayed significant correlations. In a separate study the ICA demonstrated no significant practise effect over the duration of the study.

**Discussion:** The ICA can support clinicians by aiding accurate diagnosis of MCI and AD and is appropriate for large-scale screening of cognitive impairment. The ICA is unbiased by differences in language, culture, and education.

## Introduction

Neurodegenerative disorders, including dementia and Alzheimer's Disease (AD), continue to represent a major social, healthcare and economic burden, worldwide (1). AD is the most common type of dementia with mild cognitive impairment (MCI) being a pre-dementia condition with a prevalence ranging from 16 to 20% of the population in those between 60 and 89 years old (2). Of patients suffering from MCI, 5–15% progress to dementia every year (3). These diseases remain underdiagnosed or are diagnosed too late, potentially resulting in less favourable health outcomes as well as higher costs on healthcare and social care systems (4).

In anticipation of disease-modifying treatments for MCI and AD (5, 6), the importance of early diagnosis has become increasingly pressing (7). There is accumulating evidence that early detection provides cost savings for health care systems and is an achievable goal (8, 9), and accurate patient selection for disease-modifying treatments is cost-effective and will improve clinical outcomes (5).

Timely identification and diagnosis is considered to be key to tackling dementia offering multiple benefits to patients, families and caregivers, healthcare providers, as well as society as a whole (10). If achieved, early detection can aid the deceleration of the progression of the disease (8). Early disease identification can consolidate preventative efforts through modifiable lifestyle factors to limit the progression of the disease (11).

The available neuroimaging and fluid biomarkers of neurodegeneration are not easily accessible or scalable as health services cannot provide them routinely (5). As a result, neuropsychological assessments remain the mainstay of dementia diagnosis. Current routinely used neuropsychological assessments are invariably paper-based, language and education-dependent, have ceiling effects and require substantial clinical time to administer. They lack reliability in preclinical stages of dementia (12, 13), and are prone to classification errors (14). Such tests are typically subject to practise effects (15), which may lead to incorrect estimates of age-related changes. Invariably, these tests require administration by a clinician and therefore are not appropriate for remote measurements or longitudinal monitoring.

Computerised cognitive tests constitute promising tools for the early detection of clinically relevant changes in MCI and AD sufferers (7, 16–18). Computer and mobile device-based tests have been investigated to some extent, with several showing good diagnostic accuracy entering clinical use (13, 19, 20). Devices such as smartphones and tablets offer a nearly unlimited number of applications that can be combined for the comprehensive assessment of cognition and brain health in community-dwelling users.

Digital biomarkers can help identify and monitor subtle cognitive changes in individuals at risk of developing dementia (21). Furthermore, such tests are able to benefit from advanced analytics for more accurate and personalised results. Artificial intelligence (AI) and machine learning are being increasingly used for applications in Dementia research (7). These tools have also been applied to detection of dementia using electronic health records and neuroimaging data (22–24). Recent evidence suggests that three-quarters of older people are open to new technology to make an accurate and early diagnosis and would agree to using computer or smartphone tasks that monitor day-to-day life (25).

Computerised cognitive assessments have primarily been designed to mirror traditional pen-and-paper tests, ultimately missing the opportunity to obtain additional diagnostically useful cognitive information (26). Moreover, current computerised cognitive assessments have been struggling with facing trade-offs in terms of sensitivity and specificity, required effort and adherence, while others have been criticised for testing their technology with younger adults (27).

Significantly, not much attention has been paid to personalised care in order to adapt to the individual's cognitive and functional characteristics, such as offering tailored information to the needs of the patients (28).

We developed the Integrated Cognitive Assessment (ICA) which is a 5-min, self-administered, computerised cognitive assessment tool based on a rapid categorisation task which is independent of language (29, 30). The ICA primarily tests information processing speed (IPS) and engages higher-level visual areas in the brain for semantic processing, i.e., distinguishing animal vs. non-animal images (29), which is the strongest categorical division represented in the human higher-level visual cortex (31). IPS underlies many areas of cognitive dysfunction (32, 33) and is one of the key subtle, early changes that is slowed down in pre-symptomatic Alzheimer's disease (34). This is because the speed with which an individual performs a cognitive task is not an isolated function of the processes required in that task, but also a reflection of their ability to rapidly carry out many different types of processing operations.

In the case of the ICA, these operations include transferring visual information through the retina to higher level visual areas i.e., sensory speed, processing the image representation in the visual system to categorise it into animal or non-animal (i.e., cognitive speed), and then translating this into a motor response i.e., motor speed.

The ICA employs AI to detect cognitive impairment. We aimed to evaluate the generalisability of the ICA in detecting cognitive impairment in MCI and mild AD patients. We recruited participants from two cohorts in different continents. We hypothesise that the AI-model employed for ICA can be generalised across demographically different patient populations.

To measure the convergent validity of the ICA with standard of care cognitive tests we compared the ICA with the Montreal Cognitive Assessment (MoCA) and Addenbrooke's Cognitive Examination (ACE). We investigated the level of education bias between the cognitive assessments.

We also report the effects of repeated exposure to the test in healthy participants (learning bias).

## Materials and Methods

### The ICA Test Description

The ICA test is a rapid visual categorisation task with backward masking, and has been described in detail in previous publications (29, 30). The test takes advantage of the human brain's strong reaction to animal stimuli (35–37). One hundred natural images (50 of animals and 50 of not containing an animal) of various levels of difficulty are selected and are presented to the participant in rapid succession as shown in the Supplementary Figure 1.

Images are presented at the centre of the screen at 7° visual angle to the participant. In some images the head or body of the animal is clearly visible to the participants, which makes it easier to detect. In other images the animals are further away or otherwise presented in cluttered environments, making them more difficult to detect.

The strongest categorical division represented in the human higher level visual cortex appears to be that between animals and inanimate objects (38, 39). Studies also show that on average it takes about 100 to 120 ms for the human brain to differentiate animate from inanimate stimuli (36, 40, 41). Following this rationale, each image is presented for 100 ms followed by a 20 ms inter-stimulus interval (ISI), followed by a dynamic noise mask (for 250 ms), followed by subject's categorisation into animal vs. non-animal. Shorter periods of ISI can make the animal detection task more difficult and longer periods reduce the potential use for testing purposes as it may not allow for the detection of less severe cognitive impairments. The dynamic mask is used to remove (or at least reduce) the effect of recurrent processes in the brain (42, 43). This makes the task more challenging by reducing the ongoing recurrent neural activity that could artificially boost the subject's performance; it further reduces the chances of learning the stimuli. For more information about rapid visual categorisation tasks refer to Mirzaei et al., (44).

Grayscale images are used to remove the possibility of colour blindness affecting participants' results. Furthermore, colour images can facilitate animal detection solely based on colour (45, 46), without fully processing the shape of the stimulus. This could have made the task easier and less suitable for detecting mild cognitive deficits.

The ICA test begins with a different set of 10 trial images (5 animal, 5 non-animal) to familiarise participants with the task. If participants perform above chance (>50%) on these 10 images, they will continue to the main task. If they perform at chance level (or below), the test instructions are presented again, and a new set of 10 introductory images will follow. If they perform above chance in this second attempt, they will progress to the main task. If they perform below chance for the second time the test is restarted.

Backward masking: To construct the dynamic mask, following the procedure in (47), a white noise image was filtered at four different spatial scales, and the resulting images were thresholded to generate high contrast binary patterns. For each spatial scale, four new images were generated by rotating and mirroring the original image. This leaves us with a pool of 16 images. The noise mask used in the ICA test was a sequence of eight images, chosen randomly from the pool, with each of the spatial scales to appear twice in the dynamic mask.

### Reference Pen-and-Paper Cognitive Tests

#### Montreal Cognitive Assessment

MoCA is a widely used screening tool for detecting cognitive impairment, typically in older adults (48). The MoCA test is a one-page 30-point test administered in approximately 10 min.

#### Addenbrooke's Cognitive Examination (ACE)

The ACE was originally developed at Cambridge Memory Clinic (49, 50). ACE assesses five cognitive domains: attention, memory, verbal fluency, language and visuospatial abilities. On average, the test takes about 20 min to administer and score.

ACE-R is a revised version of ACE that includes MMSE score as one of its sub-scores (51). ACE-III replaces elements shared with MMSE and has similar levels of sensitivity and specificity to ACE-R (52).

### Study Design

We aimed at studying the ICA across a broader spectrum of geographical locations with differences in language and culture to test the generalisability of the ICA. For analytical purposes we combined participants from two cohorts in order to study the ICA in one demographically diverse population.

See Table 1 for a summary of the demographic characteristics of recruited participants.

**Table 1:** Summary of demographic information, and cognitive test scores of recruited participants.

				**Age**		**Education years**		**MoCA**		**ACE**		**ICA Index**	
**Cohort**	**Diagnosis**	**Count**	**Female (%)**	**Mean**	**SD**	**Mean**	**SD**	**Mean**	**SD**	**Mean**	**SD**	**Mean**	**SD**
Cohort 1	Healthy	33	57.6	63.6	6.7	14.2	4.7	25.9	3.0	92.1	6.7	66.1	7.7
	MCI	27	55.6	66.0	7.0	14.2	5.7	23.7	2.9	87.3	7.3	57.8	8.1
	mild AD	13	46.2	69.8	9.4	11.2	4.0	16.6^a^	5.8	68.5	14.3	41.6	14.4
Cohort 2	Healthy	62	54.8	68.5	7.6	14.3	4.2	28.3	1.8	95.7	3.1	63.7	8.7
	MCI	53	43.4	71.5	7.9	12.5	2.7	23.5	2.9	84.1	6.9	54.7	11.9
	mild AD	42	50.0	71.6	7.4	13.3	3.2	20.2	3.0	76.1	8.1	46.9	15.5
Combined	Healthy	95	55.8	66.8	7.6	14.3	4.4	27.5	2.6	94.4	4.9	64.5	8.4
	MCI	80	47.5	69.6	8.0	13.1	4.0	23.6	2.9	85.2	7.2	55.7	10.8
	mild AD	55	49.1	71.2	7.9	12.8	3.5	19.3	4.1	74.3	10.3	45.6	15.3

a *The minimum MoCA score was 8; this participant had a low number of education years (3 years), ACE score of 49 and mini mental state examination score of 17*.

#### Cohort 1

73 (33 Healthy, 27 MCI, 13 mild AD) participants completed the ICA test, MoCA and ACE-R in the first assessment. The participants were non-English speakers, with instructions for the cognitive assessments provided in Farsi. MCI and mild AD patients and were recruited from neurology outpatients at the Royan Research Institute, Iran during a single visit. Healthy participants were recruited by a number of means including from patient companions, local advertisements at the royan research institute and family friends. All assessment scales were carried out by a trained psychologist or a study doctor.

All diagnoses were made by a consultant neurologist (Z.V) according to diagnostic criteria described by the working group formed by the National Institute of Neurological and Communicative Disorders and Stroke (NINCDS) and the Alzheimer's Disease and Related Disorders Association (ADRDA) and the National Institute on Ageing and Alzheimer's Association (NIA-AA) diagnostic guidelines (53). All study participants had previously had an MRI-head scan, blood tests, and physical examination as part of the diagnostic procedure.

The study was conducted at Royan institute. The study was conducted according to the Declaration of Helsinki and approved by the local ethics committee at Royan Institute. The inclusion exclusion criteria are listed in the Supplementary Material. Informed written consent was obtained from all participants.

#### Cohort 2

157 (62 Healthy, 53 MCI, 42 mild AD) participants with a clinical diagnosis completed the ICA test, MoCA and ACE-III. The ICA test was taken on an iPad. Participants of age-range 55–90 were included in this study. The study was conducted at 6 NHS sites in the UK and participants were recruited from NHS memory clinics. Healthy participants were recruited from spouses and carers of patients presenting to the memory clinics and through service user advocate groups following researcher presentations in the respective NHS trusts. All assessment scales were administered by trained Psychologists or Nurses. Ethics approval was received from the London Dulwich Research Ethics Committee. Informed written consent was obtained from all participants.

All diagnoses were made by a memory clinic consultant psychiatrist according to the same diagnostic criteria as in Cohort 1. The diagnostic procedure included an MRI-head scan, blood tests and physical examination for all participants. The eligibility criteria are listed in the Supplementary Material. One additional inclusion criterion for Cohort 2 required an ACE-III score of >=90 for healthy participants. Cognitive assessments were performed either in the clinic, or via at home visits. Approximately 51% of assessments were conducted via home visits and 49% in the clinic in a single visit.

Inclusion criteria were common for both cohorts and refer to individuals with normal or corrected-to-normal vision, without severe upper limb arthropathy or motor problems that could prevent them from completing the tests independently (see Supplementary Material). For each participant, information about age, education and gender was also collected. Informed written consent was obtained from all participants.

Spectrum bias, whereby the subjects included in the study do not include the complete spectrum of patient characteristics in the intended use population (54) has been avoided in Cohort 1 and Cohort 2 by recruiting participants according to a sampling matrix and at the mild stage of Alzheimer's Dementia. Therefore, the ICA performance metrics reported in this study are relative to detecting cognitive impairment in a population with less severe impairment.

### Analysis Methods

#### Accuracy, Speed and Summary ICA Index Calculation

The raw data from the ICA is composed of reaction time and categorisation accuracy on the images. This data was used to calculate summary features such as overall accuracy, and speed using the same methodology as described previously (29, 30).

Accuracy is defined as follows:

$$\begin{array}{l} Accuracy=\frac{Number\;of\;correct\;categoristions}{Total\;number\;of\;images}\times 100 \end{array}$$

Speed is defined based on participant's response reaction times in trials they responded correctly:

$$\begin{array}{l} Speed=\min\left[100,\;100e^{-\frac{mean\;correct\;RT}{1025}+0.341}\right] \end{array}$$

A summary ICA Index, is calculated as follows:

$$\begin{array}{l} ICA\;Index=\left(\frac{Speed}{100}\times \frac{Accuracy}{100}\right)\times 100 \end{array}$$

The ICA Index describes the raw test result, incorporating speed and accuracy, the two main elements of the ICA test.

#### ICA AI Model

The AI model utilises inputs from accuracy and speed of responses to the ICA rapid categorisation task (with the ICA Index as an input feature), as well as age, and outputs an indication of likelihood of impairment (AI probability) by comparing the test performance and age of a patient to those previously taken by healthy and cognitively impaired individuals. The AI model is able to achieve an improved classification accuracy relative to using any single feature from the ICA test.

A probability threshold value of 0.5 was used to convert the AI probability to the AI prediction of healthy or cognitively impaired (MCI/mild AD). The AI probability was also converted to a score between 0 and 100 using the following equation:

$$\begin{array}{l} ICA\;score=\left(1-AI\;probability\right)\times 100 \end{array}$$

**Figure 1:**
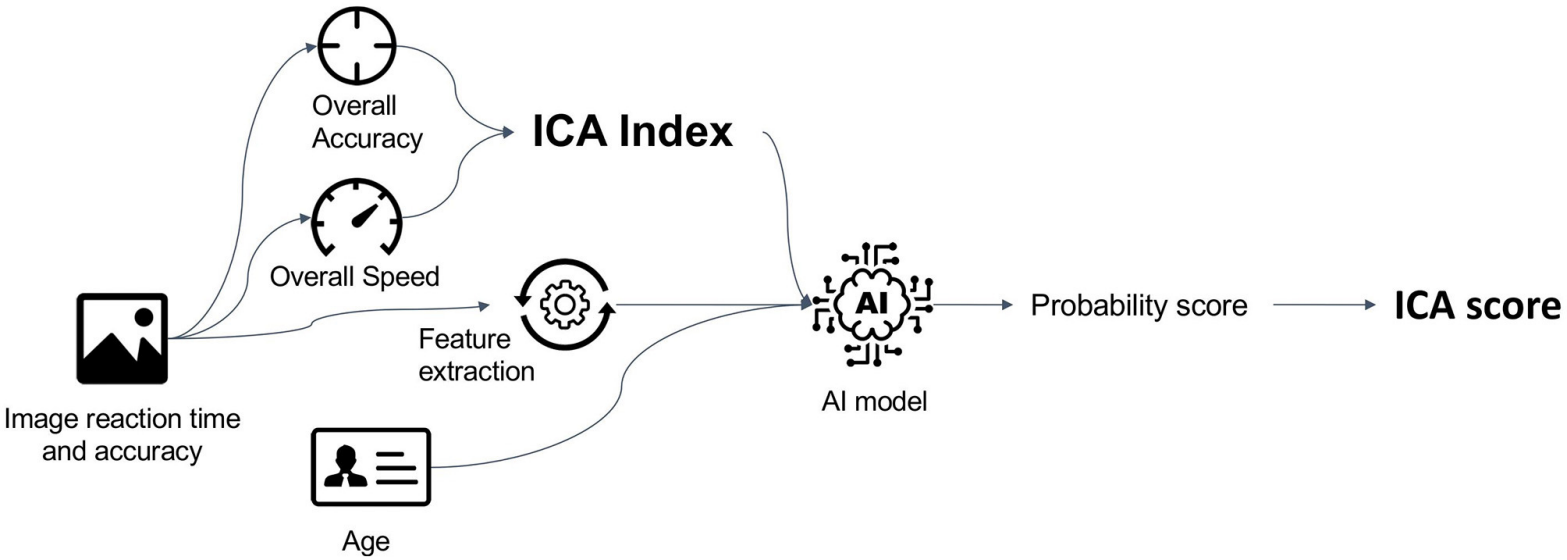
Data features from the ICA test, as well as age are used as features to train the AI model. The trained model is able to give predictions on new unseen data. The AI model outputs a probability score between 0 and 1, which is converted to an ICA Score.

The ICA AI model used in this study was a binary logistic regression machine learning model which is a supervised linear classifier implemented on Python scikit-learn with stochastic gradient descent learning (55). The algorithm's task is to learn a set of weights from a regression model that maps the participant's ICA test results and demographics to the classification label of healthy or cognitively impaired (Figure 1). An example results-page from the ICA, showing the ICA Score obtained from the AI model, as well as the informative features of the ICA test such as the accuracy, speed and ICA Index are shown in Supplementary Figure 2.

The ICA's prediction on each participant was obtained using leave-one-out-cross validation on the data from Cohort 1 and Cohort 2. In this method the dataset was repeatedly split into two non-overlapping training and testing sets. The training set was used to train the model and the test set to test the performance of the classifier. In the leave-one-out method only one instance was placed in the test set, with the remaining data points used for training. This was done iteratively for all data points, providing an AI probability score for each participant.

The combined results were used to calculate overall metrics (receiver operating curve area under the curve (ROC AUC), sensitivity, specificity) for the classifier by comparing the ICA AI prediction to clinical diagnosis. The sensitivity or true positive rate is the proportion of actual positives–i.e., impaired- that are identified as such, whilst the specificity, or true negative rate is the proportion of actual negatives–i.e., healthy–that are identified as such.

The ICA AI prediction was also compared to MoCA, ACE to obtain percentage agreement values between these cognitive tests. Single cut-off values were used to obtain predictions for MoCA (score of ≥26 for healthy) and ACE (score of ≥90 for healthy).

### Generalisability of the ICA, and Impact of Training Data Size on Classification Performance

In order to test the generalisability of the ICA, data from Cohort 1 was used to train the AI model and was tested on data from Cohort 2, and vice versa. The number of data points used to train the AI model can significantly impact the performance of the model on the testing dataset. To investigate this, subsets of the data from one cohort were used for training through random sampling. For each training size a model was trained and tested on all the data from the other cohort. We varied the size of the training data from 3 data points to training with all the data from each cohort.

### Assessment of ICA Practise Effect

To investigate the practise effect related to the ICA, 12 healthy participants (range of 26–73, mean 48.2 standard deviation 17.1 years) took 78 tests on a regular basis (936 tests in total). Participants were trained remotely, with assistance provided as needed to initiate the test platform. Thereafter all tests were taken independently at a time and place of the participant's choosing. Reminders via electronic correspondence were sent periodically to users to encourage adherence.

The time taken for users to complete the 78 tests was 96.8 days on average (standard deviation of 31.7 days). Participants self-administered the ICA remotely, on Apple iPhone devices.

### Baseline Characteristics of Participants

In total 230 participants (Healthy: 95, MCI: 80, mild AD: 55) were recruited into Cohort 1 and Cohort 2. Participant demographics and cognitive test results are shown in Table 1. Participants were recruited based on a sampling matrix in order to minimise age, gender, and education year differences across the three arms.

Healthy participants did have a lower age compared to mild AD participants. This is reflective of the lower prevalence of young mild AD patients in the general population. There was no significant statistical difference in education years between any of the groups after Bonferroni correction for multiple comparisons. For complete table of *t*-test *p*-values for age and education years see in the Supplementary Table 1.

Due to the balanced recruitment, there was no significant difference across genders in any of the cognitive tests (See in Supplementary Figure 3). The ICA also did not show a significant difference in score between those with 0–11 years education, compared to those with 12 years of education or more (Supplementary Figure 3b). In contrast there was a statistically significant increase in MoCA score for mild AD participants of higher education, while ACE scores were higher for those with higher education years in Healthy and MCI participants (Table 2).

**Table 2:** Mean and standard deviation of ICA, MoCA, and ACE scores broken down by education years.

	**Healthy**			**MCI**			**Mild AD**		
	**≤11 education years** **(mean ± SD)**	**12+ education years** **(mean ± SD)**	**Paired *t*-test** ***p*-value**	**≤11 education years** **(mean ± SD)**	**12+ education years** **(mean ± SD)**	**Paired *t*-test** ***p*-value**	**≤11 education years** **(mean ± SD)**	**12+ education years** **(mean ± SD)**	**Paired *t*-test** ***p*-value**
ICA Index	62.7 ± 9.2	65.5 ± 7.8	0.129	55.5 ± 12.3	55.8 ± 9.7	0.908	43.5 ± 15.2	47.1 ± 15.4	0.385
MoCA	27.3 ± 3.1	27.6 ± 2.3	0.625	22.7 ± 3.3	24.2 ± 2.3	0.021	17.4 ± 4.2	20.7 ± 3.5	0.003^*^
ACE	92.1 ± 7.1	95.6 ± 2.8	0.001^*^	81.7 ± 7.2	87.8 ± 6	<0.001^*^	70.5 ± 10.3	77 ± 9.5	0.019

*P-value of t-test between those with 0–11 years education, and those with 12+ years of education, for each cognitive test across the three arms.*

* *indicates significant difference after Bonferroni correction for multiple comparisons*.

This trend was also illustrated in correlation analysis. The ICA displayed a Pearson r correlation of 0.17 with education years (*p* = 0.01), which is considerably smaller than that of MoCA (*r* = 0.34, *p* < 0.0001) and ACE (*r* = 0.41, *p* < 0.0001) which displayed significant correlations.

### ICA Convergent Validity With MoCA and ACE

**Figure 2:**
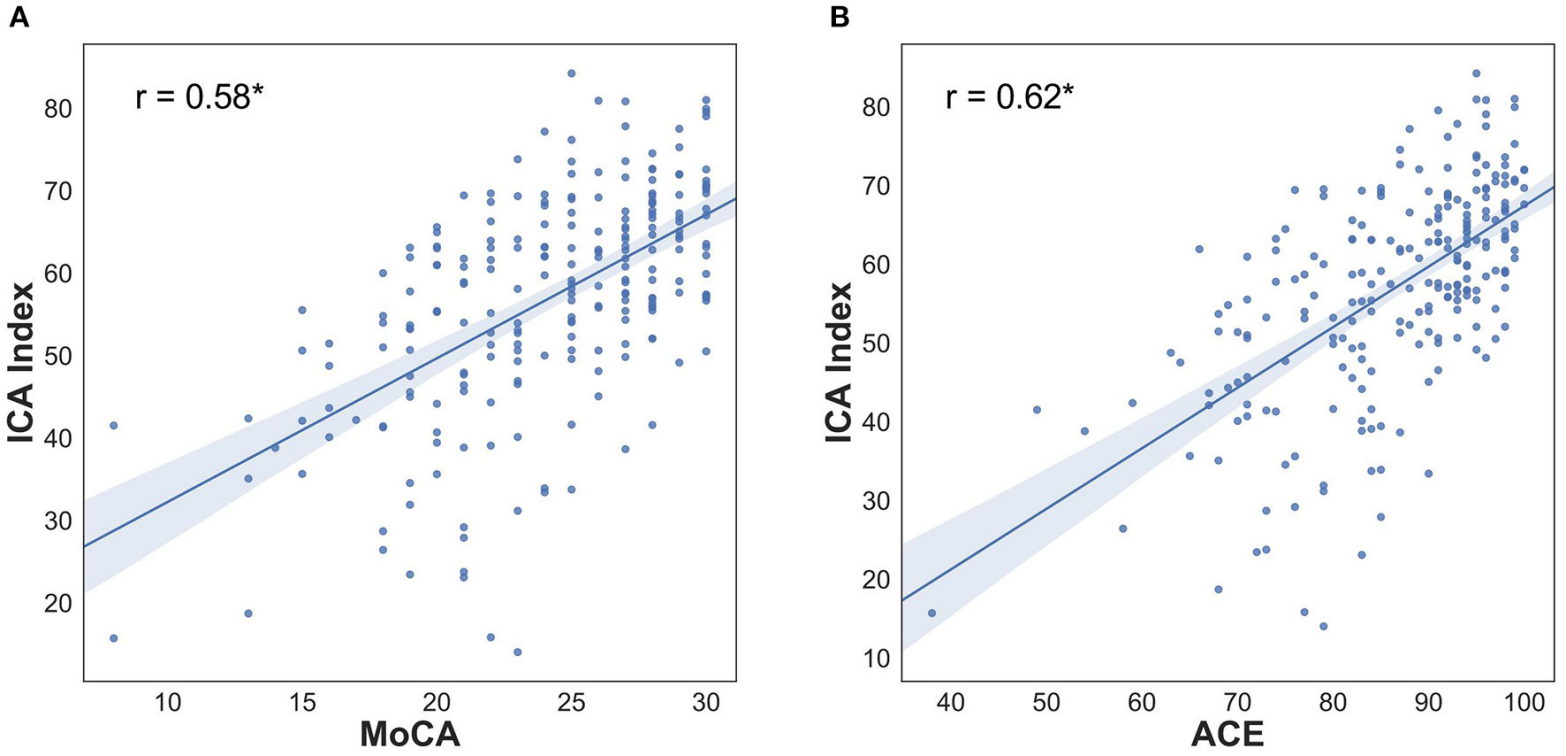
ICA Index correlation with MoCA and ACE **(A)** Pearson correlation: 0.58, *p* < 0.0001, ICA Score Pearson correlation with MoCA is 0.58, *p* < 0.0001 **(B)** Pearson correlation 0.62, *p* < 0.0001; ICA Score Pearson correlation with ACE is 0.56, *p* < 0.0001. For breakdown of correlation with ACE subdomains see Table 3.

The statistically significant Pearson correlation of 0.62 with ACE and 0.58 with MoCA demonstrates convergent validity of ICA with these cognitive tests. The scatterplot of ICA with MoCA and ACE is shown in Figure 2. A ceiling effect was observed for MoCA and ACE as a high proportion of healthy participants have maximum test scores, something not observed for the ICA. However, none of the tests were observed to have floor effects, including in the mild AD group.

The breakdown of the ICA Index correlation with the individual cognitive domains as measured by ACE is shown in Table 3. In all domains, the Pearson correlation is >0.3, with the strongest correlation obtained with the Memory and Fluency component and a weakest correlation with language.

**Table 3:** Correlation of the ICA with cognitive domains of ACE.

**ACE domain**	**Pearson correlation**	**Pearson *p*-value**
Memory	0.53	*p* < 0.0001
Attention	0.41	*p* < 0.0001
Fluency	0.53	*p* < 0.0001
Language	0.31	*p* < 0.0001
Visuospatial	0.48	*p* < 0.0001

### Speed and Accuracy of Processing Visual Information

The breakdown of speed and accuracy by age and diagnosis is shown in Table 4. Within Healthy participants, there is a strong negative correlation between age and accuracy (Pearson *r* = −0.4, *p* < 0.0001); similarly, for MCI participants (Pearson *r* = −0.4, *p* < 0.001). However, for mild AD participants there is no correlation between their accuracy and age (Pearson *r* = 0.07, *p* = 0.58). Analysis did not reveal a significant difference in speed with age within the three groups.

**Table 4:** Mean speed and accuracy on the ICA test, by age category and diagnosis.

		**Speed**		**Accuracy**	
	**Age**	**Mean**	**STD**	**Mean**	**STD**
Healthy	<70	76.3	8.4	85.9	7.6
MCI		73.7	11.1	78.9	9.6
mild AD		70.7	18.0	64.8	17.6
Healthy	≥70	78.3	8.6	80.6	7.6
MCI		71.3	14.0	74.5	11.8
mild AD		69.6	16.6	65.6	15.3

Healthy participants have a significantly higher accuracy compared to MCI and mild AD participants in the under 70 age category (*t*-test *p* < 0.001 after Bonferroni correction for multiple comparisons). MCI participants also had a significantly higher accuracy compared to mild AD in the under 70 age category (*t*-test *p* < 0.001). In the over 70 age category, healthy participants had significantly higher accuracy compared to mild AD (*t*-test *p* < 0.001). The other pairwise comparisons by age category and diagnosis were not found to be significant after Bonferroni correction.

Overall, across all ages the Cohen's D between healthy and MCI participants for accuracy is 0.72 (*p* < 0.0001), and between healthy and mild AD participants it is 1.46 (*p* < 0.0001). Cohen's D value is 0.41 (*p* = 0.006) for speed (Healthy vs. MCI across all ages), compared to 0.52 (*p* = 0.001) for healthy vs. mild AD participants. The Cohen's D for MoCA is 1.43 for Healthy vs. MCI (*p* < 0.0001), and 2.37 (*p* < 0.0001) for Healthy vs. mild AD. While Cohen's D measures the effect size, in the next section we present results on comparing the diagnostic accuracy of ICA and MoCA in relation to clinical diagnosis.

Prior to the commencement of the 100 image ICA test, participants are shown a set of trial images for training purposes. If users perform adequately well on the trial images, they proceed to the main test, however if they perform below chance then the trial images are re-shown to participants. We observed that the number of attempts required by participants before proceeding on to the main test is itself a strong predictor of cognitive impairment. Among Healthy participants 88% completed the trial images on their first attempt compared to 61% of MCI participants, and 44% of mild AD participants (See in Supplementary Figure 4).

Furthermore, within each group, those who required more than one attempt to progress onto the main test scored lower than those who did not, and they tended to be older participants (see in Supplementary Table 2), indicating within-group cognitive performance variation.

### ICA Accuracy in Detecting Cognitive Impairment

**Figure 3:**
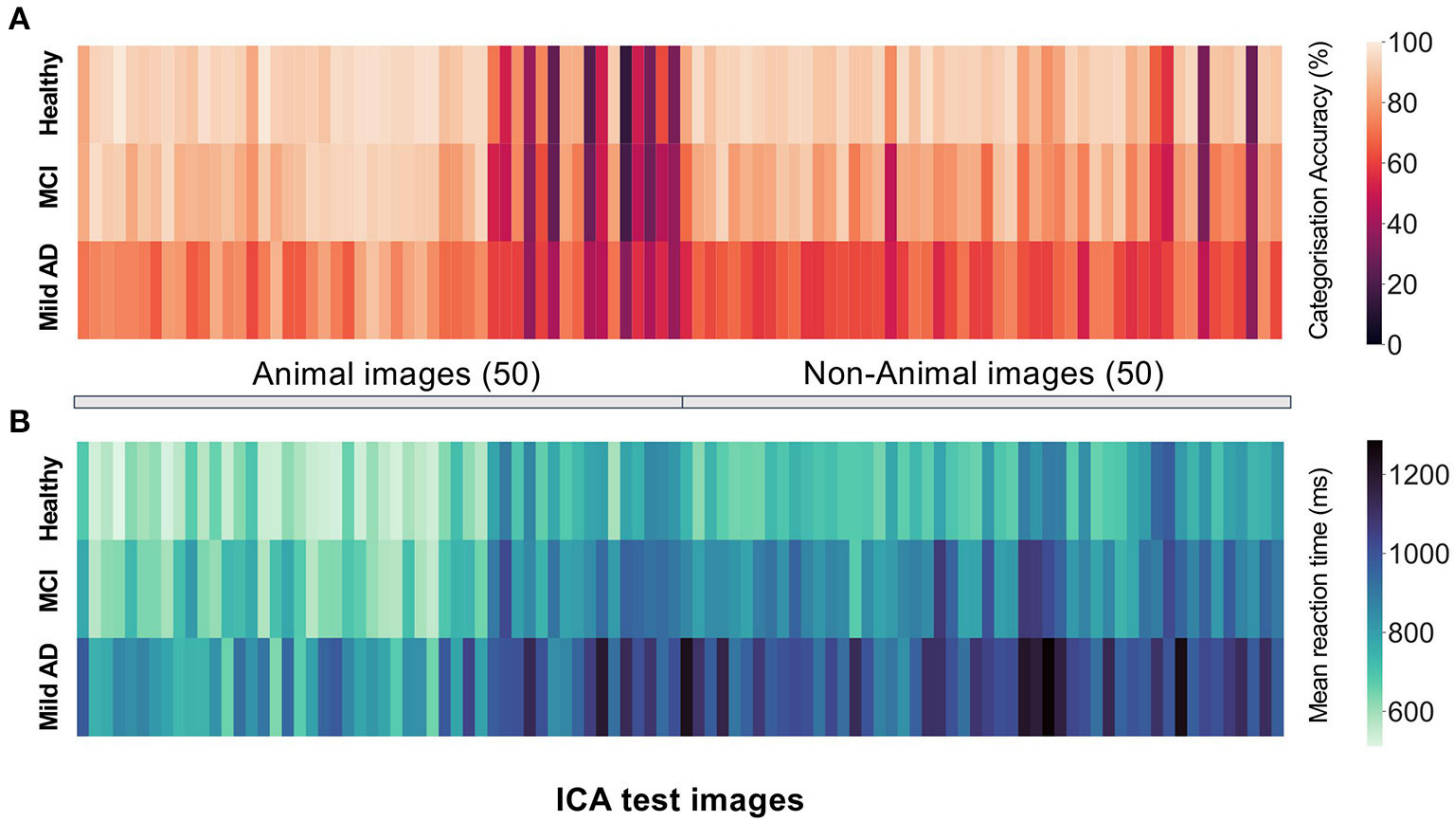
The mean **(A)** categorisation accuracy **(B)** reaction time for participants of each diagnosis group for each image on the ICA test. The first 50 blocks represent performance on the animal images, and the second 50 blocks represent performance on the non-animal images. In the actual ICA test the order of the images is randomised.

The raw data from the ICA test consist of categorisation accuracy and reaction time for each of the 100 images on the test. In Figure 3, the average accuracy and reaction time per image has been visualised as a heat map for each group to show how healthy and impaired participants (MCI and Mild-AD) perform on the ICA test. The sequence of images shown to users during the test is randomised, therefore for ease of comparison the images have been ordered by their category of animal, non-animal.

Healthy participants display significantly higher categorisation accuracy (Figure 3A) and significantly lower mean reaction time (Figure 3B). However, the varying difficulty of individual images results in a spread of categorisation accuracy for all three groups.

The participants' age, ICA Index, and features based on the speed and accuracy of responses to the categorisation task extracted from the ICA test were used to train a binomial logistic regression model for classification of healthy vs. MCI/mild AD participants.

Leave one out cross-validation (LOOCV–see methods for full description) was used to obtain a probability of impairment and predictions for each participant. This method ensures the maximum amount of data is used for training the model, while ensuring a separation between the training and test data points. A threshold value of 0.5 was used as the cut-off between healthy and impaired participants.

**Figure 4:**
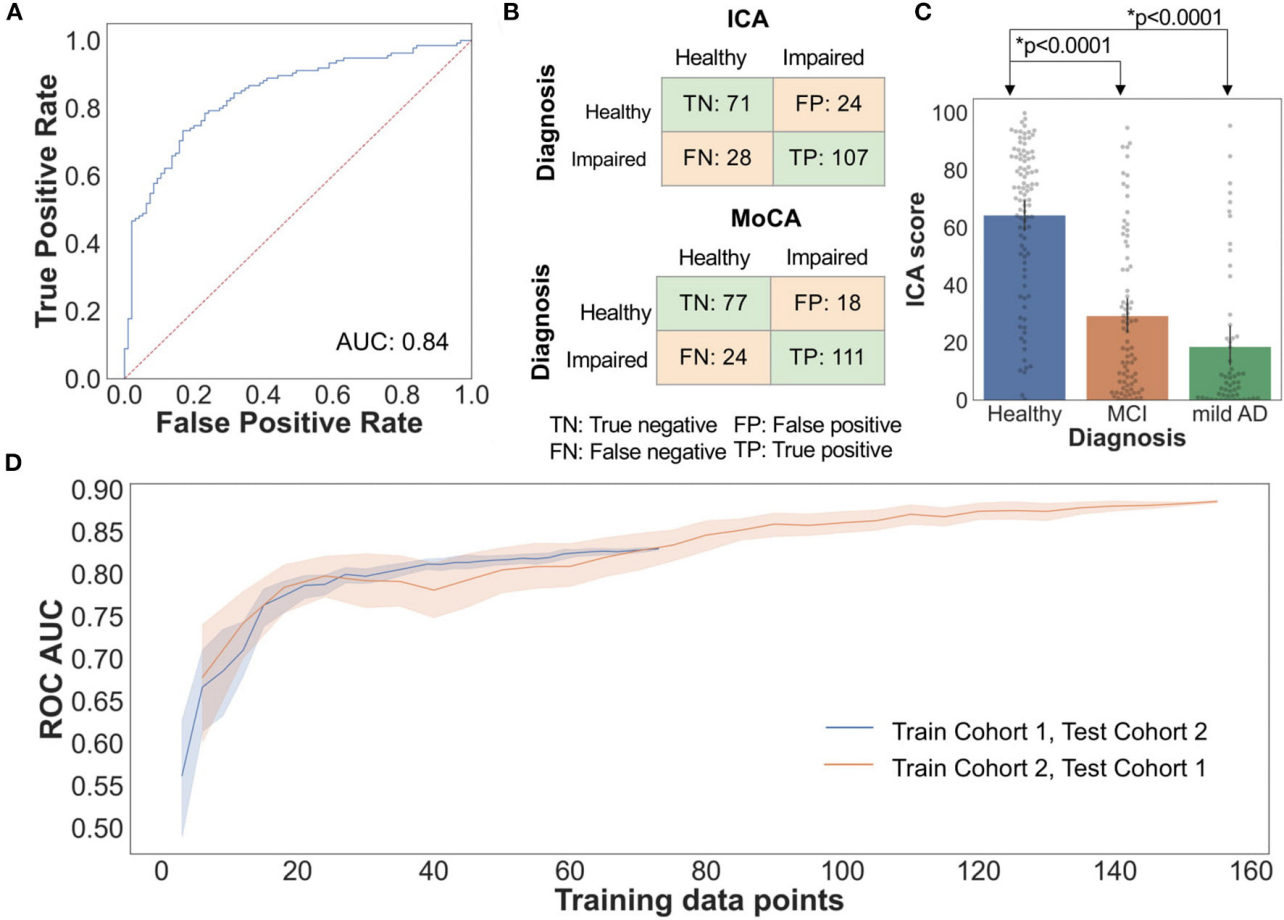
**(A)** Healthy vs. Impaired ROC: AI classification performance by LOOCV **(B)** The confusion matrix for the ICA and MoCA, comparing the prediction of the cognitive tests with clinical diagnosis. **(C)** Bar plot with 95% confidence interval of ICA Score for healthy, MCI, and mild AD, with all data points overlaid on the graph. *t* test *p*-value comparing Healthy-MCI, and healthy–mild AD ICA score is also shown **(D)** ROC AUC vs. training data size. The shaded area represents 95% CI as each training subset was selected randomly 20 times from the whole study data.

Figures 4A,B shows the ROC and confusion matrix for distinguishing healthy from impaired (MCI/mild AD) participants. The AUC is 0.84 for distinguishing healthy from impaired with a sensitivity of 79% and specificity of 75%.

For the AI score a higher value is indicative of being cognitively healthy, and a lower score is indicative of potential cognitive impairment. Healthy participants have significantly higher ICA score compared to MCI and mild AD participants (Figure 4C).

A comparison of the classification performance between the ICA and MoCA is shown in Table 5. A score of ≥26 is the MoCA cut-off for healthy participants, and lower than 26 MoCA cut-off for cognitive impairment, as outlined by Nasreddine et al., (48). Optimal cut-offs for MoCA have been found to vary by ethnicity and language (56), however for consistency across the two cohorts 26 was used as the cut-off score. Likewise, the ICA AI model cut-off was consistent in both cohorts for fair comparison.

**Table 5:** Classification performance metrics for ICA and MoCA, with 95% confidence intervals (CI). The AUC for ICA is calculated based on the continuous probability output score.

**Cognitive test**	**Classification**	**AUC (95% CI)**	**Sensitivity (95% CI)**	**Specificity (95% CI)**
ICA	Healthy vs. Impaired	0.842 (0.791, 0.893)	79.3 (72.4, 86.1)	74.7 (66.0, 83.5)
ICA	Healthy vs. MCI	0.814 (0.749, 0.878)	76.2 (66.9, 85.6)	74.7 (66.0, 83.5)
ICA	Healthy vs. mild AD	0.883 (0.823, 0.944)	83.6 (73.9, 93.4)	74.7 (66.0, 83.5)
MoCA	Healthy vs. Impaired	0.816 (0.765, 0.868)	82.2 (75.8, 88.7)	81.1 (73.2, 88.9)
MoCA	Healthy vs. MCI	0.768 (0.705, 0.831)	72.5 (62.7, 82.3)	81.1 (73.2, 88.9)
MoCA	Healthy vs. mild AD	0.887 (0.84, 0.934)	96.4 (91.4, 100.0)	81.1 (73.2, 88.9)

A recent systematic review of pen and paper tests showed that across 20 studies, for healthy vs. MCI, MoCA displayed AUC of 85.1%, specificity of 74.6%, and sensitivity of 83.9% (57). A direct comparison of this type is not provided for ACE, as this cognitive test was used as an inclusion criterion for healthy participants in Cohort 2, and hence by default it would have a specificity of 100% for healthy participants from that study.

Table 6 demonstrates the percent agreement between the ICA and MoCA/ACE prediction. In both cases the overall percent agreement is >73%, with the positive percent agreement (where both tests predicted impaired) higher than the negative percent agreement (where both tests predicted healthy). It should be noted that agreement here does not imply correct prediction, as the cognitive tests themselves can misclassify participants.

**Table 6:** Percent agreement between ICA and MoCA, ACE, with 95% confidence intervals.

	**Positive percent agreement** **(95% CI)**	**Negative percent agreement** **(95% CI)**	**Overall percent agreement** **(95% CI)**
ICA and MoCA prediction	77.5 (70.3, 84.7)	69.3 (60.3, 78.3)	73.9 (68.2, 79.6)
ICA and ACE prediction	81.4 (74.2, 88.6)	66.7 (58.1, 75.2)	73.9 (68.2, 79.6)

The ICA AI model has been made explainable by utilising representative, and clinically relevant data from clinical and research studies for training and testing of the model. An inherently more understandable learning algorithm (logistic regression) has been used in favour of more complex “black box” models such as deep learning. An example results page from the ICA is shown in the Supplementary Figure 2. In addition to the AI output (ICA Score), the overall accuracy, speed, ICA Index and performance during the test is displayed. As shown in the results presented here, these additional metrics are highly correlated with diagnosis, clinically informative, and help explain the AI output (ICA score), providing supporting evidence to aid the clinician in diagnosis.

### ICA AI Model Generalisability

Figure 4D demonstrates how changing the training sample size impacts the ICA AI model classification accuracy as measured by ROC AUC. Randomly selected subsets of data from Cohort 1 were used as training data, and tested on all of Cohort 2 data, and vice-versa. With small training data sets there is significant fluctuation in performance with wide confidence intervals. Increasing the number of training data points increases the ROC AUC. Furthermore, this analysis demonstrates the generalisability of the ICA AI-model and therefore the ICA test score across two demographically similar populations.

### Assessment of ICA Practise Effect on Remote Monitoring of Cognition

To assess practise effect, we recruited healthy participants to control for the risk of fluctuating or progressively lower test scores in cognitively impaired individuals. The mean ICA Index of the 12 healthy participants, with the 95% confidence interval is shown in Supplementary Figure 5. The one-way ANOVA *p*-value obtained was 0.99, showing no significant practise effect for the participants who completed the ICA test 78 times over a period of 96.8 days on average.

## Discussion

In this study we show that the ICA establishes convergent validity with standard-of-care cognitive assessments such as MoCA and ACE. In contrast to these tests the ICA is not confounded by varying levels of education. Similarly, in previous studies conducted in MS patients and healthy controls (174 participants in total) and another study with 436 participants on individuals aged 19–98, ICA was shown to have no significant correlation with education years (29, 30).

The ICA can generalise across populations without the need for collection of population-specific normative data. This was demonstrated by the ability of the ICA to detect patterns of cognitive impairment that are common across cohorts of different cultural and demographic characteristics. Conventional pen and paper and computerised tests require renorming and validation in different languages in order to be validated, requiring collection of culture-specific normative data before a test can be used in populations with different demographic characteristics. Both are prerequisites for large population deployment and risk-based screening in primary care.

We show that the ICA demonstrates no practise effect in healthy participants. As patients with MCI can improve, remain stable, or decline cognitively over time, it is vital that they are monitored regularly for changes in their cognitive status, which could alter diagnosis and management of their care (3). MCI monitoring can enable a timely diagnosis and treatment. Available interventions can improve the trajectory of symptoms and the family's ability to cope with them, and thus change the experience of the course of dementia (58).

The diagnostic accuracy of the ICA, while not perfect, is comparable to results reported for MoCA, and make the ICA suitable as a screening test given its significantly shorter duration and other advantages over existing tests. In light of the recent FDA approval of the disease modifying drug aducanumab, the need for a device capable of screening a wide population of at-risk individuals is heightened.

In this study we have demonstrated the sensitivity of the ICA in detecting cognitive impairment at the early stages of cognitive impairment. Within the impaired category we have only recruited those with MCI or mild AD, with the majority being MCI across the two cohorts. Therefore, the ICA performance metrics are relative to detecting cognitive impairment in such a population. The expectation is that a higher accuracy would be obtained on those with more progressed levels of cognitive impairment.

The conventional method for classification in cognitive assessments is defining a single cut-off value from the test score. This may lead to diagnostic misclassification as single, static cut-offs cannot sufficiently account for factors such as the patient's age, education level, cognitive reserve and premorbid IQ. The high-dimensional dataset generated by the ICA, as well as the ability to incorporate demographic features, provide more parameters that can be optimised, enabling a classifier to find the optimum classification boundary in higher dimensional space. The ICA's classification accuracy can be further improved over time by training on additional data to update the AI model.

The ICA AI model can also be expanded to include and analyse additional patient data such as medication, sleep, and other lifestyle factors along with other biomarker data to improve its accuracy and support the development of predictive models of neurodegeneration.

IPS data captured by the ICA show different signature patterns between healthy, MCI, and mild AD patients and generate a rich enough dataset to train the ICA AI model to distinguish healthy from cognitively impaired individuals. Subtle changes in IPS can remain undetected, unless rigorously assessed. We are not aware of another cognitive test that quantifies IPS changes to the degree of ms.

The use of AI in decision making, particularly for diagnostic decisions in healthcare, requires a level of explainability from the model which can be used to understand the important factors which led to its output (59). This level of explainability can give clinicians confidence in the model, protect against bias and can be used to improve the performance of the system over time. This is in contrast to high accuracy ‘black box' models that offer limited interpretability of results and therefore prohibit their use in clinical practise.

The ICA results are automatically calculated, eliminating the clinical time required for test interpretation while minimising transcription errors. Test results can be integrated in electronic health records or research databases, an important capability at the intersection between primary and secondary care. The ICA's ease of use and short duration can improve pre-screening and accelerate participant selection in clinical trials.

Study limitations include a relatively lower recruitment of young participants with mild AD, and mild AD participants with higher education years. However, this is reflective of the lower prevalence of young mild AD patients in the general public. Test-retest data have not been captured in this study. We have previously reported that high test-retest reliability (Pearson *r* > 0.91) was obtained for the ICA (29, 30).

Fluid or molecular biomarker sub-typing to determine amyloid positivity for MCI participants has not been carried in this study, due to lack of data availability. The MCI group, however, reflects the heterogeneity MCI diagnoses in memory clinics. We plan to correlate fluid biomarker positivity with the ICA in future studies.

Remote cognitive assessment is becoming increasingly important, particularly as health services cannot accommodate regular patient attendance to memory services for progression monitoring or response to treatments. The COVID-19 pandemic has accelerated this pressing need and guidelines for the implementation of partly or fully remote memory clinics have recently been published (16). Digital cognitive and functional biomarkers are essential in order to enable this. We report a proof-of-concept capability of the ICA for the remote measurement of cognitive performance. Our findings suggest that this 5-min test can identify broad cognitive impairments across different stages of impairment. Further validation is required for remote administration in MCI and mild AD patients.

In summary the ICA can be used as a digital cognitive biomarker for the detection of MCI and AD. Furthermore, the ICA can be used as a high frequency monitoring tool both in the clinic and potentially remotely. The employment of AI modelling has the potential to further enhance its performance but also to personalise its results at an individual patient level across geographic boundaries.

## Data Availability Statement

The raw data supporting the conclusions of this article will be made available by the authors, without undue reservation.

## Ethics Statement

The studies involving human participants were reviewed and approved by the local ethics committee at Royan Institute (Cohort 1) and by the London Dulwich Research Ethics Committee (Cohort 2). The patients/participants provided their written informed consent to participate in this study.

## Author Contributions

CK and MHM: contributed equally as co-first authors to this work. S-MK-R, CK, and DA: conception and design of the study. MK, HK, and ZV: data collection. MHM, CK, and S-MK-R: data analysis. CK, MHM, PA, and S-MK-R: manuscript writing. All authors contributed to the article and approved the submitted version.

## Conflict of Interest

S-MK-R serves as the Chief Scientific Officer at Cognetivity Ltd. CK serves as the Chief Medical Officer at Cognetivity Ltd and Principal Investigator on NIHR and Industry-funded clinical trials. MHM and PA are employed by Cognetivity Ltd. DA has received research support and/or honoraria from Astra-Zeneca, H. Lundbeck, Novartis Pharmaceuticals, Biogen, and GE Health, and served as paid consultant for H. Lundbeck, Eisai, Heptares, and Mentis Cura. The remaining authors declare that the research was conducted in the absence of any commercial or financial relationships that could be construed as a potential conflict of interest.
